# Promoting Seed Germination of Some Plant Species by Rhamnolipid Produced by *Pseudomonas aeruginosa*

**DOI:** 10.1155/2024/7137413

**Published:** 2024-04-25

**Authors:** Zeena Ghazi Faisal

**Affiliations:** Department of Biology, College of Education, Al-Iraqia University, Baghdad, Iraq

## Abstract

With growing environmental concerns and a growing world population, there is an interest in developing cheap, efficient, and environmentally friendly substances that benefit humanity. Microbial surfactants are nontoxic, biodegradable bioproducts that provide sustainable solutions in agricultural applications due to their many benefits over their synthetic counterparts. Hence the crucial importance of scientific research to understand the effect of microbial surfactants on plant development. The present study aimed to assess the effect of rhamnolipid produced by *Pseudomonas aeruginosa* on seed germination of wheat (*Triticum aestivum*), barley (*Hordeum vulgare*), okra (*Abelmoschus esculentus*), onion (*Allium cepa*), and lettuce (*Lactuca sativa*) under laboratory conditions. The results showed that *P. aeruginosa* was capable of producing 3.83 g/L of viscous, honey-colored rhamnolipid, which was capable of lowering the surface tension to 30 ± 0.33 mN/m. Different concentrations of rhamnolipid ranging from 0.25 to 1.00 g/L were assessed, with distilled water acting as a control. After treatment of seeds, results showed that applying 0.25 g/L of rhamnolipid can significantly increase seed germination to 100% on the fourth day of sowing okra and lettuce, and on the fifth day of sowing onion seeds, compared to control groups that recorded 60%, 50%, and 55%, respectively. In wheat and barley seeds, applying rhamnolipid can protect seeds from pathogenic fungi while delaying their germination to 60% and 70% on the third day of sowing, while 90% and 100% have been reported in the control groups. Thus, this biological molecule demonstrates promising results in enhancing seed germination of the studied species by protecting them from phytopathogens and then aiding plant growth.

## 1. Introduction

Agricultural production poses a significant challenge for all countries in terms of meeting the rising demand for human food. Recently, new strategies have been developed to improve agriculture production and achieve sustainability through the use of eco-friendly and cost-effective natural products such as biosurfactants [1, 2]. The surface active compounds created by bacteria, yeasts, and fungi are low-toxic and biodegradable substances used in the manufacture of agricultural products as an alternative to synthetic surfactants [3, 4]. Additionally, they have a variety of beneficial impacts on seeds, including antifungal, antibacterial, and antiviral activities, improve the bioavailability of nutrients for plants, and play an important role in enhancing the microbial-plant interaction beneficial to plants [5, 6]. Furthermore, biosurfactants have the potential as bioremediation agents to improve the health of soil systems by increasing the solubility of harmful and remaining pesticides, which can make them available for biodegradation by other microorganisms [7].

Seed germination is thought to be the most delicate stage of a plant's life cycle [8]. Pre-sowing seed stimulation is a process of seed priming that accelerates seed germination by supplying the initial dose of nutrients for germination and providing broad-spectrum protection through enhancing resistance to plant pathogens and supporting plant local and systemic immunity [4]. Because of the sustainable approach, coating seeds with natural products has gained worldwide interest. In this sense, priming can potentially support rapid seed germination and ensuing growth.

Biosurfactants-based natural stimulation is considered an economical and accurate method that can significantly increase the vitality of planted seeds by protecting them from phytopathogen attack, stimulating plant immunity, facilitating the uptake of biogenic elements by plants, such as phosphorus, reducing the initial dose of fertilizers, and protecting seeds from harmful compounds present in the soil. [5, 6, 9]. Furthermore, biosurfactants can enhance the availability of minerals in soil by increasing the solubility of ionic nutrients and enhancing the distribution of micronutrients and minerals in the soil, leading to increased uptake by plants [4, 9].

The purpose of this study was to determine the effect of rhamnolipid produced by *Pseudomonas aeruginosa* on seed germination and growth of several plant species, including wheat (*Triticum aestivum*), barley (*Hordeum vulgare*), okra (*Abelmoschus esculentus*), onion (*Allium cepa*), and lettuce (*Lactuca sativa*), in the laboratory.

## 2. Material and Methods

### 2.1. Extraction of Rhamnolipid

For the extraction of partially purified rhamnolipid, *P. aeruginosa* was grown in mineral salt medium MSM (pH7), composed of (g/L): KH_2_PO_4_ (1.0), K_2_HPO_4_ (1.0), NaCl (2.0), CaCl_2_ (0.05), MgSO_4_.7H_2_O (0.5), FeCl_3_ (0.002), yeast extract (0.1), olive oil (4%), and NH_4_NO_3_ (1%), then incubated for 9 days at 30°C in a 150 rpm shaker incubator. The fermented culture was centrifuged at 25°C for 10 minutes at 12.000 rpm. The cell-free supernatant was acidified to pH 2 and stored at 4°C overnight.

At room temperature (25°C) and in a volumetric flask, equal volumes of the cell-free supernatant and chloroform : methanol (2 : 1 v/v) were mixed well. By using a separation funnel, the organic phase was collected and dried in glass Petri dishes at 40°C [10]. Crude rhamnolipid was recovered as a viscous dark honey-coloured product that was stored at 4°C for further studies.

### 2.2. Seed Sample Collection and Seeds Sterilization

Seeds of wheat, barley, okra, onion, and lettuce plants that widely consumed in our societies were selected. Viable pest-free seeds were obtained from the Iraqi Ministry of Agriculture, State Board for Seed Testing and Certification. They were rinsed thoroughly with sterile distilled water and then immersed in a 3% sodium hypochlorite solution for 4 minutes with constant stirring, thereafter washed five times with sterile distilled water to remove any effect of the disinfectants [11].

### 2.3. Assessment of Rhamnolipid Activity in Promoting Seed Germination and Growth

The influence of rhamnolipid on seed germination of the studied plants was determined by preparing five Petri dishes for each plant species and performing experiments on five replicates.

Different concentrations of the partially purified rhamnolipid (0.25, 0.50, 0.75, and 1.00 g/L) were prepared, and distilled water was used as a control. Then, 10 mL of these solutions were added to 0.24 millipore filter paper present at the bottom of Petri plates, spreading 10 seeds of each plant species on the filter paper of each plate, and incubating the plates at room temperature in a dark place. Seed germination was monitored at regular intervals for 5 days [12].

### 2.4. Data Analysis

For more than two groups, one-way analysis of variance (ANOVA) was performed, and *P* < 0.05 was the acceptable statistical significance.

## 3. Results and Discussion

### 3.1. Extraction of Rhamnolipid

3.83 g/L of viscous the honey-coloured product was obtained through acid precipitation and chloroform: methanol extraction method. This partially purified rhamnolipid can lower the surface tension to 30 ± 0.33 mN/m. The extract was used to estimate its potential to enhance seed germination and growth in the laboratory.

Many studies have demonstrated that acid precipitation and the chloroform: methanol extraction method was the most efficient technique for obtaining biosurfactant from the producing bacteria. Adebajo et al. [13] extracted rhamnolipid from *P. putida* using acid precipitation and different solvents (ethyl acetate, acetone, dichloromethane, and chloroform : methanol (2 : 1 v/v)) and discovered that chloroform: methanol yielded the most, followed by acetone, dichloromethane, ethyl acetate, and acid precipitation gave the least yield. Tripathi et al. [14] used acid precipitation and chloroform: methanol for the extraction of biosurfactants from *S*. *maltophilia* IITR87, *O. anthropi* IITR07, *M*. *esteraromaticum* IITR47, *P*. *mendocina* IITR46, and *P*. *aeruginosa* IITR48, yielded 1.146, 0.981, 0.804, 0.510, and 0. 360 g/L, respectively. In contrast, Sumiardi et al. [10] used acid precipitation and a chloroform: methanol solvent extraction technique to extract the biosurfactant from *B. subtilis* strain ANSKLAB03, and obtained 3.24 g/L.

### 3.2. Assessment of Rhamnolipid Activity in Promoting Seed Germination and Growth

Results indicated the obvious effect of rhamnolipid on promoting seed germination of studied plants in plates treated with various concentrations of rhamnolipid compared to the untreated control groups. In general, rhamnolipid had a significant effect on germination rates (*p* < 0.05). At a concentration of 0.25 g/L, the rhamnolipid solution displayed the highest stimulation of seed germination. This amount can also protect seeds from fungal infections. In contrast, increased rhamnolipid concentrations can inhibit seed germination. However, adding rhamnolipid before germination can have a favorable effect in protecting seeds against microbial attack as biosurfactants have antifungal, antibacterial, and antiviral properties [5, 6].

**Figure 1:**
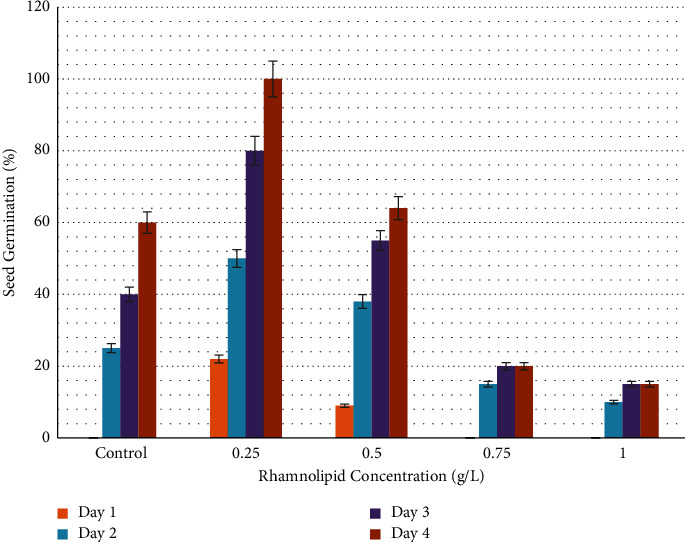
Germination rate of okra seeds (*Abelmoschus esculentus*) treated with different concentrations of rhamnolipid. A 0.25 g/L of rhamnolipid can significantly increase seed germination from the first day of sowing.

**Figure 2:**
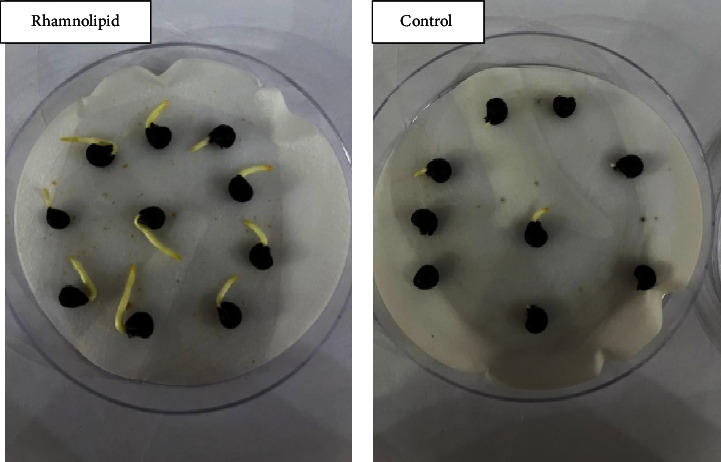
The effect of rhamnolipid produced by *P. aeruginosa* on okra seed germination after four days of sowing.

Figure 1 illustrates the germination rate of okra seeds treated with different concentrations of rhamnolipid. After one day of seeding, 22% and 9% of okra seeds were germinated in plates containing 0.25 and 0.50 g/l of rhamnolipid, while no germination was observed in the control group and at rhamnolipid concentrations of 0.75, and 1.00 g/l. After two days of sowing, 25% of seeds were germinated in the control group, and 50%, 38%, 15%, and 10% were recorded in plates with 0.25, 0.50, 0.75 and 1.00 g/L of rhamnolipid, respectively. The germination rate increased gradually reaching 100%, 64%, 20%, and 15% on the fourth day of sowing at concentrations 0.25, 0.50, 0.75, and 1.00 g/L of rhamnolipid, respectively, while it recorded 60% in the control group. When the incubation period increased, the fungal infection began to appear in the control group, while the plates treated with different concentrations of rhamnolipid were not affected. Therefore, 0.25 g/L rhamnolipid was the best in stimulating okra seed germination and seed protection from fungal infection.  Figure 2 shows the effect of 0.25 g/L rhamnolipid on okra seed germination during four days of sowing.

**Figure 3:**
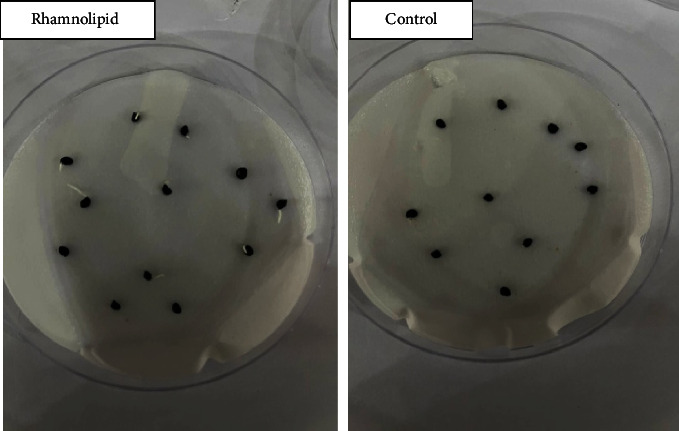
The effect of rhamnolipid produced by *P. aeruginosa* on onion seed germination after four days of sowing.

**Figure 4:**
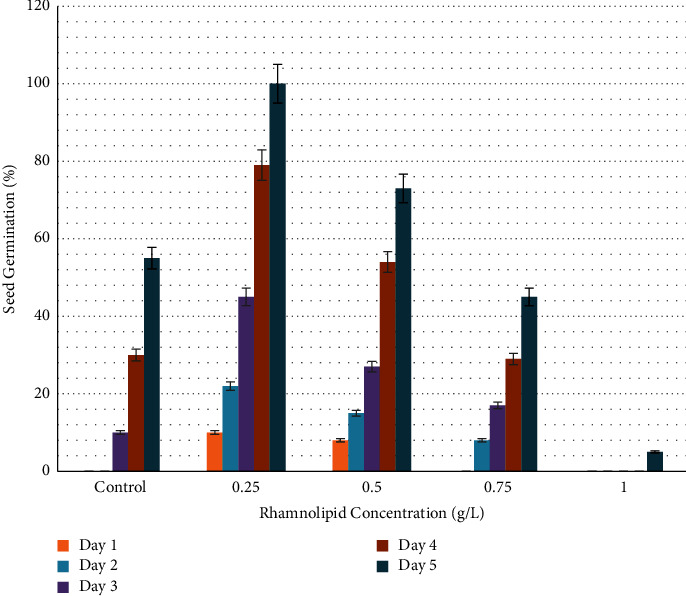
Germination rate of onion seeds (*Allium cepa*) treated with different concentrations of rhamnolipid. The concentrations of rhamnolipid 0.25 and 0.50 g/L have significant effect on seed germination from the first day of sowing.

Figure 3 shows the effect of *P*. *aeruginosa*-produced rhamnolipid at a concentration of 0.25 g/L on onion seed germination. After one day of sowing in plates containing 0.25 and 0.50 g/L of rhamnolipid, 10% and 9% of seeds germinated, respectively. No germination was seen in plates of the control group and rhamnolipid concentrations of 0.75 and 1.00 g/L. After two days of sowing, 22%, 15%, and 8% of seeds germinated in plates treated with 0.25, 0.50, and 0.75 g/L of rhamnolipid, respectively. In contrast, no signs of germination appeared in the plates of the control group and at a concentration of 1.00 g/L. The germination rate gradually increased to reach 100%, 73%, 45%, and 5% on the fifth day of sowing at the concentrations 0.25, 0.50, 0.75, and 1.00 g/L, respectively, compared to 55% in the control group. Therefore, 0.25 g/L rhamnolipid was the best in stimulating onion seed germination.  Figure 4 depicts the influence of rhamnolipid concentrations on the germination rate of onion seeds.

**Figure 5:**
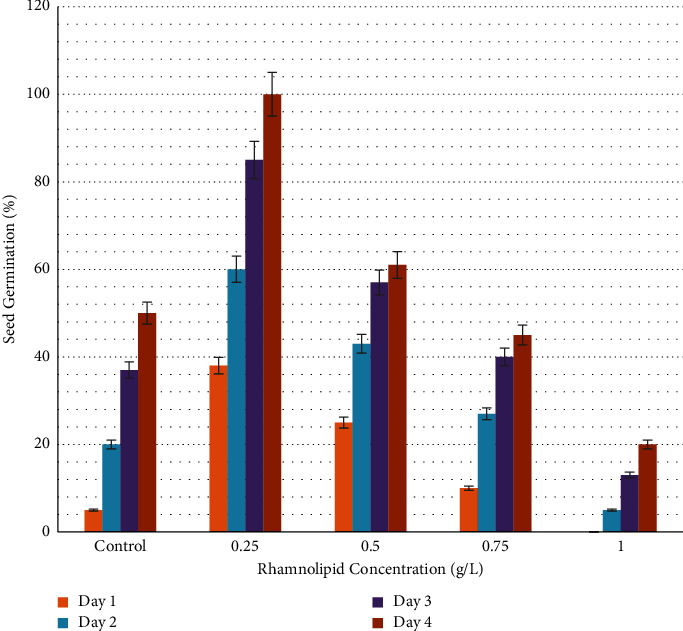
Germination rate of lettuce seeds (*Lactuca sativa*) treated with different concentrations.

**Figure 6:**
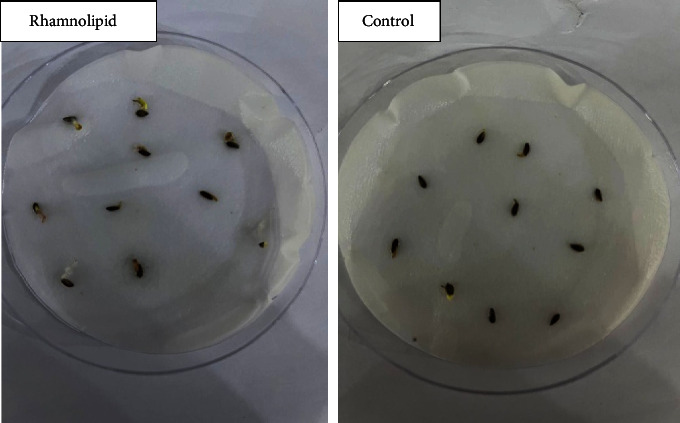
The effect of rhamnolipid produced by *P. aeruginosa* on lettuce seed germination after four days of sowing.

For lettuce seeds, germination rates of 38%, 25%, and 10% were observed after one day of sowing in plates containing 0.25, 0.50, and 0.75 g/L of rhamnolipid, respectively, compared to 5% in the control group. At 1.00 g/L of rhamnolipid, no germination was observed. After two days of sowing, the germination rates increased in the control group and plates containing 0.25, 0.50, 0.75, and 1.00 g/L of rhamnolipid, reaching 20%, 60%, 43%, 27%, and 5%, respectively. On the fourth day, all seeds germinated in the plate treated with 0.25 g/L of rhamnolipid. The remaining groups showed germination rates of 50%, 61%, 45%, and 20% in the control group and in plates containing 0.50, 0.75, and 1.00 g/L of rhamnolipid, respectively. So, 0.25 g/L rhamnolipid was the best in stimulating lettuce seed germination. Figures 5 and 6 show the influence of rhamnolipid on lettuce seed germination.

**Figure 7:**
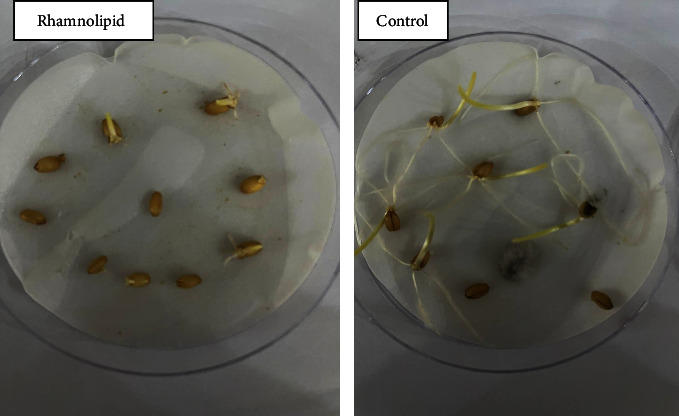
The effect of rhamnolipid produced by *P. aeruginosa* on seed germination of wheat (*Triticum aestivum*) on the third day of sowing.

**Figure 8:**
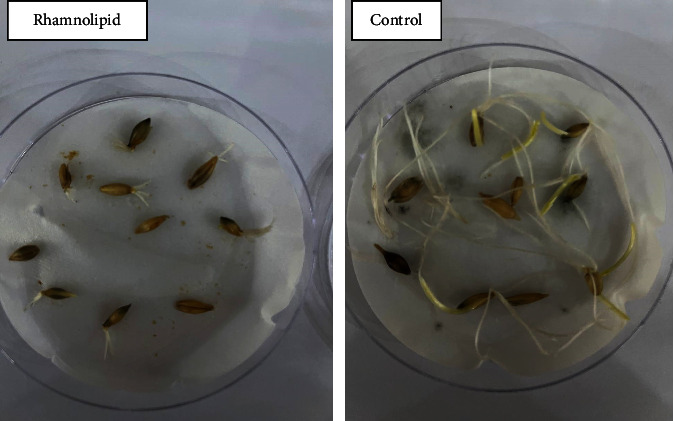
The effect of rhamnolipid produced by *P. aeruginosa* on seed germination of barley (*Hordeum vulgare*) on the third day of sowing.

**Figure 9:**
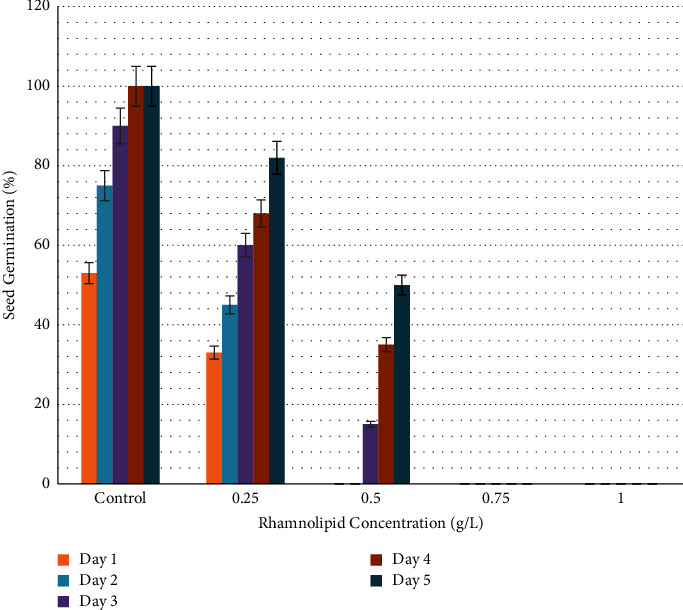
Germination rate of wheat seeds treated with different concentrations of rhamnolipid.

**Figure 10:**
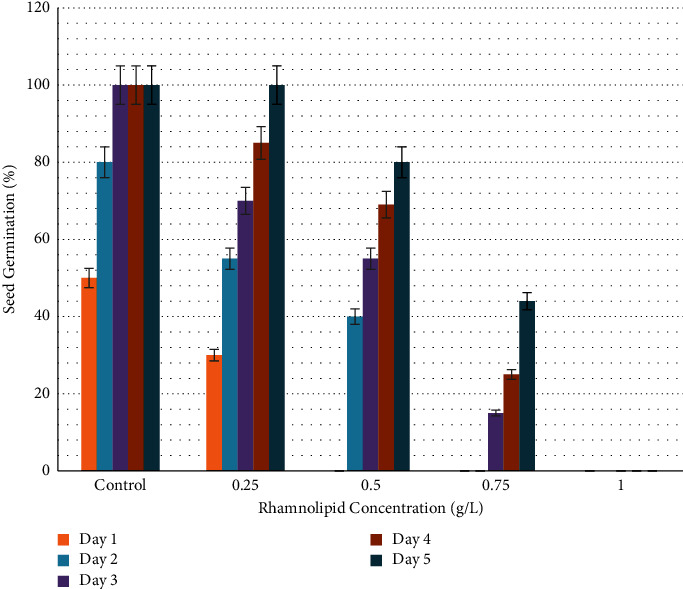
Germination rate of barley seeds treated with different concentrations of rhamnolipid.

As for wheat and barley seeds, the influence of rhamnolipid on seed germination is shown in Figures 7 and 8, respectively. After three days of sowing seeds of wheat and barley, the control groups reported 90% and 100% germination rates, respectively, with some fungal infection. Although the application of rhamnolipid successfully protected seeds from fungal infections, germination rates were reduced. Figures 9 and 10 demonstrate the germination rate of wheat and barley seeds at different rhamnolipid concentrations and in the control groups. For wheat, rhamnolipid at concentrations of 0.75 and 1.00 g/L can prevent seed germination, while 60% and 15% of seeds germinated on the third day of sowing seeds in plates treated with 0.25 and 0.50 g/L of rhamnolipid, respectively. The germination rate gradually increased without any signs of fungal infection, reaching 82% and 50% on the fifth day of sowing. As for barley seeds, germination rates of 70%, 55%, and 15% were recorded on the third day of sowing in plates treated with 0.25, 0.50, and 0.75 g/L of rhamnolipid, respectively. It rapidly increased without any fungal infection, reaching 100%, 80% and 44% on the fifth day of sowing. No germination observed at rhamnolipid concentration of 1.00 g/L. Therefore, 0.25 g/L rhamnolipid was the best in stimulating wheat and barley seed germination and seed protection from fungal infection.

These results clarify the obvious effect of *P. aeruginosa*-produced rhamnolipid in a concentration of 0.25 g/L as a minimum that can stimulate seed germination of okra, onion, and lettuce. This concentration can significantly stimulate, accelerate, and improve seed germination from the first day of incubation, while higher concentrations may reduce or delay plant germination due to potential tissue damage [15]. Also, rhamnolipid managed to protect wheat and barley seeds from fungal infection while reducing or delay seed germination compared with the control group. Indeed, the pre-treatment of seeds with biosurfactants can have a favorable effect on seed germination, as it increases the rate of water imbibition [16], or serve as a nutrient or facilitates the uptake of biogenic chemicals, such as phosphorus [12], as well as biosurfactants have antifungal, antibacterial, and antiviral properties [5, 12, 17]. Furthermore, the use of biosurfactants emerges as a safe alternative to improving agricultural soil quality by increasing nutrient bioavailability, improving the availability of hydrophobic compounds and facilitating their absorption by microbial cells, and enhancing the degradation of certain insecticides that accumulated in the agricultural soil, thus, enhancing soil fertility [9, 18].

Prabha and Jayachitra [19] reported that the presence of biosurfactant-producing *Pseudomonas* species can enhance *Oriza sativa* seed germination and shoot growth. On the other hand, Bhuyan-Pawar et al. [18] found that the use of potent biosurfactants can alleviate environmental stress, by improving the solubility of insoluble compounds, hence increasing plant productivity. Khare and Arora [20] state that the addition of biosurfactants can enhance plant-microbial interactions and improve soil properties, resulting in better disease control, plant health, and productivity.

## 4. Conclusions

As a conclusion of the current study, rhamnolipid produced by *P. aeruginosa* demonstrate variable effects in enhancing seed germination of the studied species and promising results in improving seed protection. Therefore, combined with the high ecological compatibility of these biological molecules, this can lead to new agricultural applications through its potential to be developed as green pesticides and biofertilizers, which contribute to reducing agricultural losses, more sustainable agriculture productions and augmenting organic farming in the region. Thus, field investigations of antimicrobial and biostimulant properties should be performed concurrently to determine the applicability of these potent biological molecules for increasing seed protection and fertility while alleviating environmental stress and thereby increasing plant productivity.

## Data Availability

The data used to support the findings of this study are available from the corresponding author upon request.
